# Supplementary material for: Role of higher-order exchange interactions for skyrmion stability
Source: Nat Commun. 2020 Sep 21;11:4756. doi: 10.1038/s41467-020-18473-x (PMC7506016; doi:10.1038/s41467-020-18473-x)
Supplement: Supplementary file 1 — Supplementary Information [file 41467_2020_18473_MOESM1_ESM.pdf]

Supplementary Information for:

Role of higher-order exchange interactions for skyrmion stability

Paul *et al.*

Systems	J_1/J'_1	J_2/J'_2	J_3/J'_3	J_4/J'_4	J_5/J'_5	J_6/J'_6	J_7/J'_7	J_8/J'_8	J_9/J'_9	J_{10}/J'_{10}	J_{11}/J'_{11}	B_1	Y_1	K_1
Pd/Fe/Rh(111)	13.35	−2.69	−2.84	0.62	0.46	−0.10	−0.30	−0.11	0.03	0.17	−0.06	–	–	–
	11.73	−4.31	−4.21	0.62	0.46	−0.10	−0.30	−0.11	0.03	0.17	−0.06	2.74	1.62	2.61
Pd/Fe/Ir(111)	14.40	−2.48	−2.69	0.52	0.74	0.28	0.16	−0.57	−0.21	–	–	–	–	–
	13.60	−3.28	−4.17	0.52	0.74	0.28	0.16	−0.57	−0.21	–	–	2.96	0.80	2.14
Fe/Rh/Re(0001)	9.85	0.23	−0.15	−0.22	0.27	0.05	−0.16	–	–	–	–	–	–	–
	8.85	−0.77	0.05	−0.22	0.27	0.05	−0.16	–	–	–	–	−0.39	1.00	−1.36

Supplementary Table 1 | Exchange constants obtained from DFT. Exchange constants (J_i), biquadratic (B_1), three-site four spin (Y_1) and four-site four spin (K_1) interaction constants are obtained via DFT for a fcc Pd/Fe bilayer on Rh(111), a fcc Pd/Fe bilayer on Ir(111) and a hcp Fe/Rh bilayer on Re(0001). For every system the upper line shows the exchange constants without HOI and the lower line shows the modified exchange constants J'_i upon including the HOI (cf. Table **2**) according to Eqs. (5) to (7) in the main text. Note that only the first three exchange constants need to be changed and that $J'_i = J_i$ for $i \geq 4$. The exchange constants without the HOI are taken for Pd/Fe/Rh(111) from Ref. [1], for Pd/Fe/Ir(111) from Ref. [2] and for Fe/Rh/Re(0001) from Ref. [3]. All values are given in meV. Source data are provided as a Supplementary Data 1 file.

Systems	B_1	Y_1	K_1	$\Delta E^{uudd}_{3\overline{\mathrm{K}}/4}$	$\Delta E^{uudd}_{\overline{\mathrm{M}}/2}$	$\Delta E^{3Q}_{\overline{\mathrm{M}}}$
Pd/Fe/Rh(111)	2.74	1.62	2.61	16.39	3.42	33.79
Pd/Fe/Ir(111)	2.96	0.80	2.14	8.53	2.13	34.36
Fe/Rh/Re(0001)	−0.39	1.00	−1.36	−5.31	−13.33	−21.88

Supplementary Table 2 | Higher-order exchange constants from DFT. Biquadratic (B_1), three-site four spin (Y_1) and four-site four spin (K_1) interaction constants for a fcc Pd/Fe bilayer on Rh(111), a fcc Pd/Fe bilayer on Ir(111) and a hcp Fe/Rh bilayer on Re(0001). ΔE is the energy difference between the multi-Q and the corresponding spin spiral state, i.e., $\Delta E^{uudd}_{3\overline{\mathrm{K}}/4}= E^{uudd}_{3\overline{\mathrm{K}}/4} - E^{1\mathrm{Q}}_{3\overline{\mathrm{K}}/4}$, $\Delta E^{uudd}_{\overline{\mathrm{M}}/2}= E^{uudd}_{\overline{\mathrm{M}}/2} - E^{1\mathrm{Q}}_{\overline{\mathrm{M}}/2}$, and $\Delta E^{3\mathrm{Q}}_{\overline{M}}= E^{3\mathrm{Q}}_{\overline{\mathrm{M}}} - E^{1\mathrm{Q}}_{\overline{\mathrm{M}}}$. Higher-order exchange constants are calculated using Eqs. (2) to (4) in the main text. All values are given in meV.

Systems	D_1	D_2	D_3	D_4	D_5	K	μ_s
Pd/Fe/Rh(111)	0.62	−0.04	0.04	0.00	0.05	0.17	3.2
Pd/Fe/Ir(111)	1.00	–	–	–	–	0.70	3.0
Fe/Rh/Re(0001)	0.89	–	–	–	–	−0.20	2.9

Supplementary Table 3 | Dzyaloshinskii-Moriya interaction constants, MAE and total magnetic moments obtained from DFT. Dzyaloshinskii-Moriya interaction constants for i-th neighbor spins (D_i), magnetocrystalline anisotropy energy constant (K) and total magnetic moments (μ_s) are obtained from DFT for a fcc Pd/Fe on Rh(111), a fcc Pd/Fe bilayer on Ir(111) and a hcp Fe/Rh bilayer on Re(0001). Note that a positive (negative) value of K indicates an out-of-plane (in-plane) easy magnetization direction. Values are taken for Pd/Fe/Rh(111) from Ref. [1], for Pd/Fe/Ir(111) from Ref. [2] and for Fe/Rh/Re(0001) from Ref. [3]. The magnetic moments are given in μ_B and the other parameters are given in meV. Source data are provided as a Supplementary Data 2 file.

Supplementary Note 1 | Properties of isolated skyrmions in Fe/Rh/Re(0001)

The DFT derived magnetic interaction parameters are qualitatively different for Fe/Rh/Re(0001) [3] compared to the other two systems. In particular, there is almost no frustration of exchange interactions since the nearest neighbor exchange constant J_1 is much larger than all other exchange constants (cf. Supplementary Table 2). In addition, Fe/Rh/Re(0001) exhibits an in-plane easy magnetization axis ($K < 0$), whereas the easy magnetization axis is out-of-plane for the other two film systems (cf. Supplementary Table 3).

However, the zero temperature phase diagram (Figs. 2a,b) is very similar to that of the other two film systems since the external magnetic field is applied perpendicular to the film and the value of the magnetocrystalline anisotropy constant K is only 0.2 meV, which can be compensated by magnetic fields on the order of 1 T. A transition from the spin spiral phase to the skyrmion lattice phase is found at a value of about 0.75 T and the field-polarized (ferromagnetic) phase is found at a field of above 2.6 T. In the field-polarized phase, isolated skyrmions can be stabilized. Lately, several surveys reported metastable skyrmions with in-plane magnetic anisotropy [4–9]. Note that the isolated skyrmions can also be stabilized by exchange and DMI in the presence of magnetic field by artificially setting K=0 in Fe/Rh/Re(0001) (Supplementary Figure 2). The MAE provides a positive in-plane energy component and thereby increases the skyrmion radius (compare black and blue line in Supplementary Figure 2).

When HOI are included, the skyrmions radius does not change at small fields and decreases by only a small amount at large fields (Supplementary Figure 2). These small changes are due to the small HOI constants (cf. Table 1 in the main text) and a negative sign of the biquadratic and four-site four spin constants. In Supplementary Figure 2d, we display the simulations using the value of K_1 obtained from DFT (−1.36 meV) which leads to a barrier height reduced by about 70 meV as well as for a value of $K_1 = +1.36$ meV, i.e., changing only the sign of K_1 which enhances the barrier height by about 70 meV. Note that the dependence of the barrier height on magnetic field is very similar in all cases.

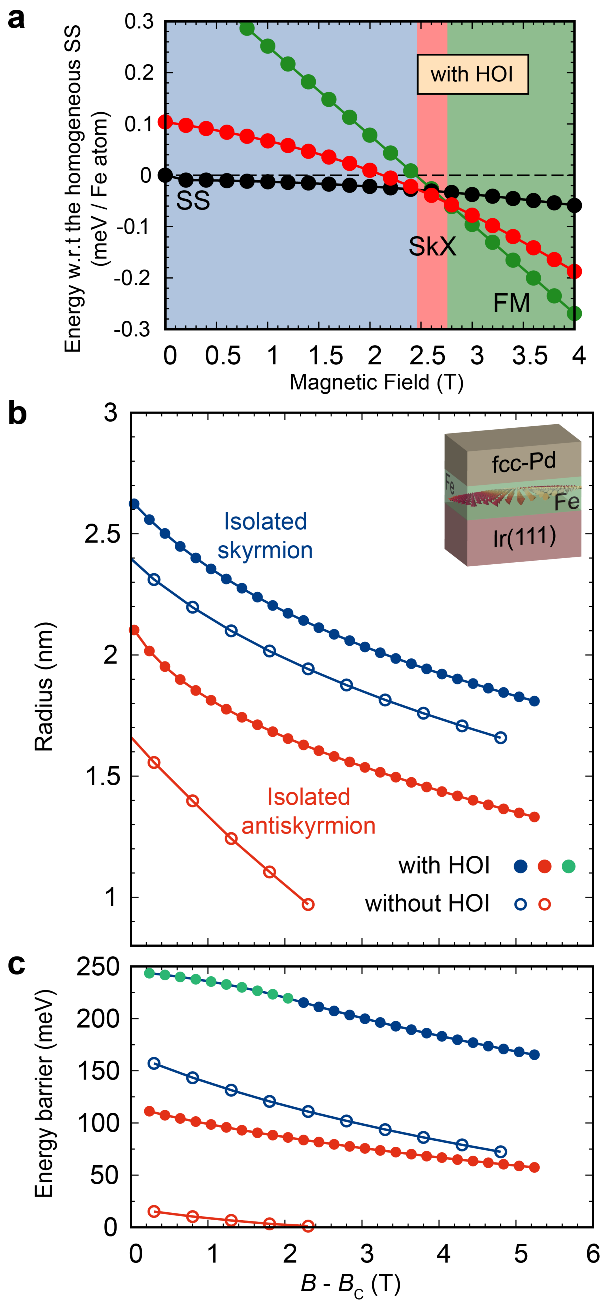

Supplementary Figure 1 | Phase diagram, radius and barrier heights for Pd/Fe/Ir(111) including HOI. a Zero temperature phase diagram of Pd/Fe/Ir(111) obtained with HOI. The energy of relaxed spin spirals (SS), skyrmion lattice (SkX) and ferromagnetic (FM) states are shown with reference to the homogeneous spin spiral (black dashed line). The SS, SkX and FM phases are denoted by blue, red and green, respectively. **b** Radius of isolated skyrmions and antiskyrmions as a function applied magnetic field with and without HOI. **c** Barrier heights of isolated skyrmions and antiskyrmions without and with HOI as a function of magnetic field. Blue and green are used to distinguish two different collapse mechanisms of isolated skyrmions with HOI. Isolated skyrmions (in presence of the higher-order terms) annihilate by a usual radial collapse mechanism (solid blue circle) from $B - B_c$= 2.2 T onwards and up to $B - B_c$=2 T, it annihilates via forming a chimera structure (solid green circle). Note that the radii and energy barriers for Pd/Fe/Ir(111) without HOI were taken from Ref. [2].

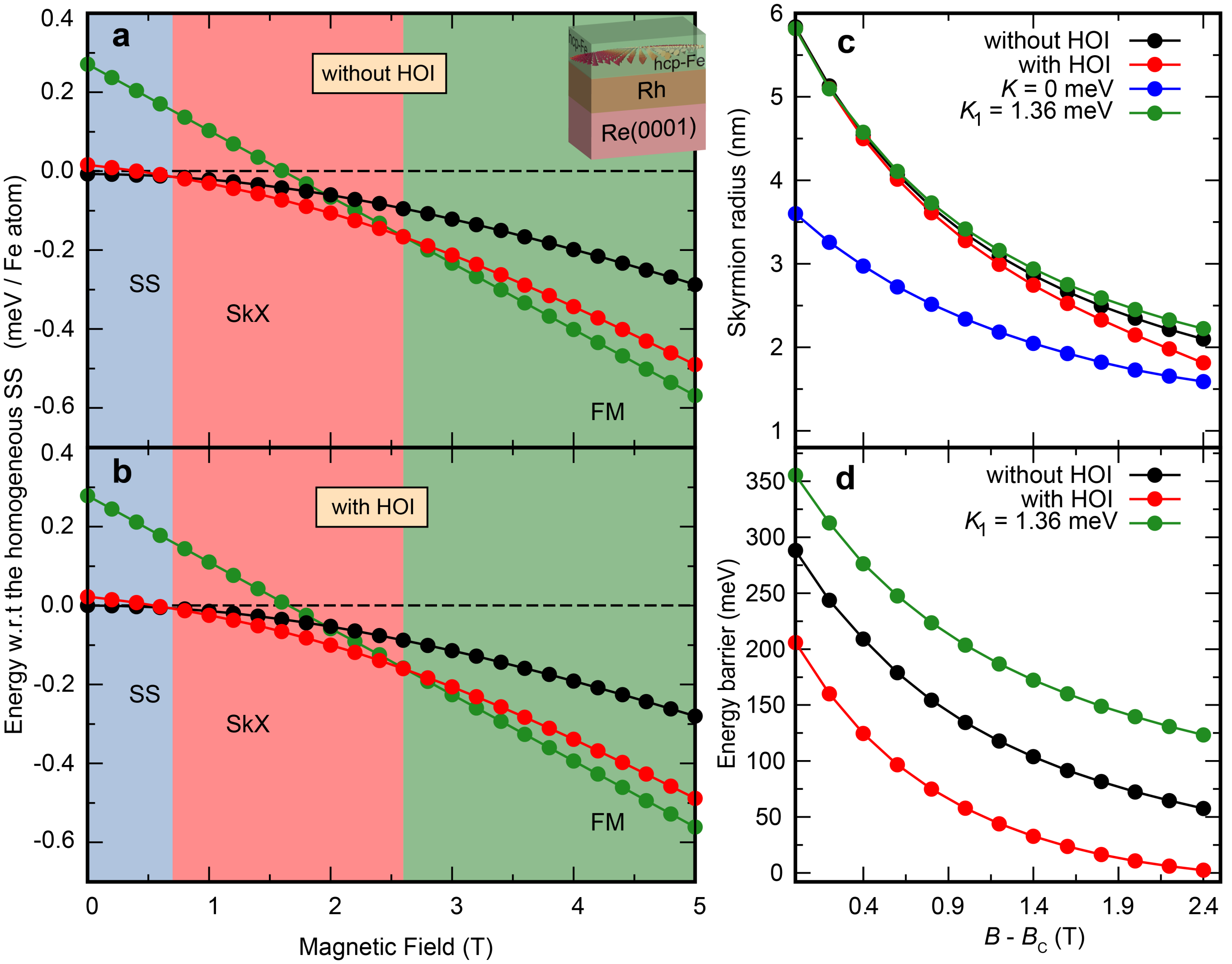

Supplementary Figure 2 | Phase diagram, radius and barrier heights for Fe/Rh/Re(0001) including HOI. a,b Zero temperature phase diagram of Fe/Rh/Re(0001) obtained without and with the HOI, respectively. The energy of relaxed spin spirals (SS), skyrmion lattice (SkX) and ferromagnetic (FM) states are shown with reference to the homogeneous spin spiral (black dashed line). The SS, SkX and FM phases are denoted by blue, red and green, respectively. **c** Radius of isolated skyrmions as a function of applied magnetic field without (black) and with (red) HOI (for HOI constants see Supplementary Table 1). Skyrmion radius by switching-off the MAE (K=0) and by setting the four-site four spin constant (K_1) to +1.36 meV, i.e., changing its sign compared to the DFT value, are shown as blue and green, respectively. **d** Barrier heights of isolated skyrmions without (black) and with (red) HOI as a function of magnetic field. Energy barriers with a positive four-site four spin constant (K_1=+1.36 meV) are shown in green.

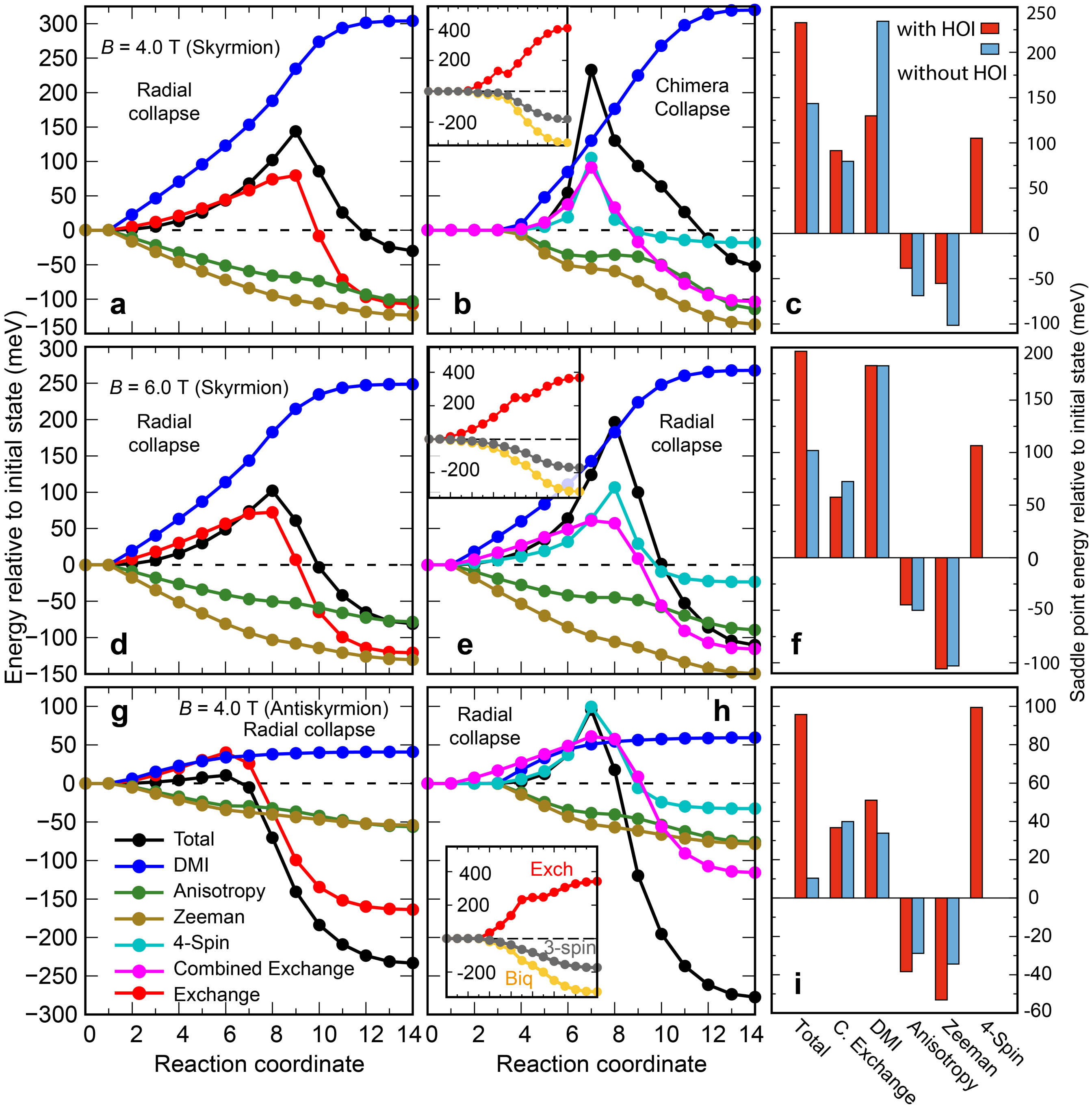

Supplementary Figure 3 | Minimum energy paths of skyrmion and antiskyrmion collapse in Pd/Fe/Ir(111). Total and individual energy contributions without (**a,d,g**) and with (**b,e,h**) the HOI along minimum energy path. Radial collapse of isolated skyrmions (**a,d**) and antiskyrmions (**g**) without the HOI. **e,h** Radial collapse path of isolated skyrmions and antiskyrmions, respectively and chimera collapse of isolated skyrmions (**b**) with the HOI. Inset of **b,e,h** The exchange, biquadratic and three-site four spin (3-spin) terms. **c,f,i** Total and individual energy decomposition at the saddle point with respect to the initial state (skyrmion/antiskyrmion) without and with the higher-order contributions. For brevity, the two-site, three-site, and four-site four spin interactions are denoted as Biq, 3-spin and 4-spin, respectively.

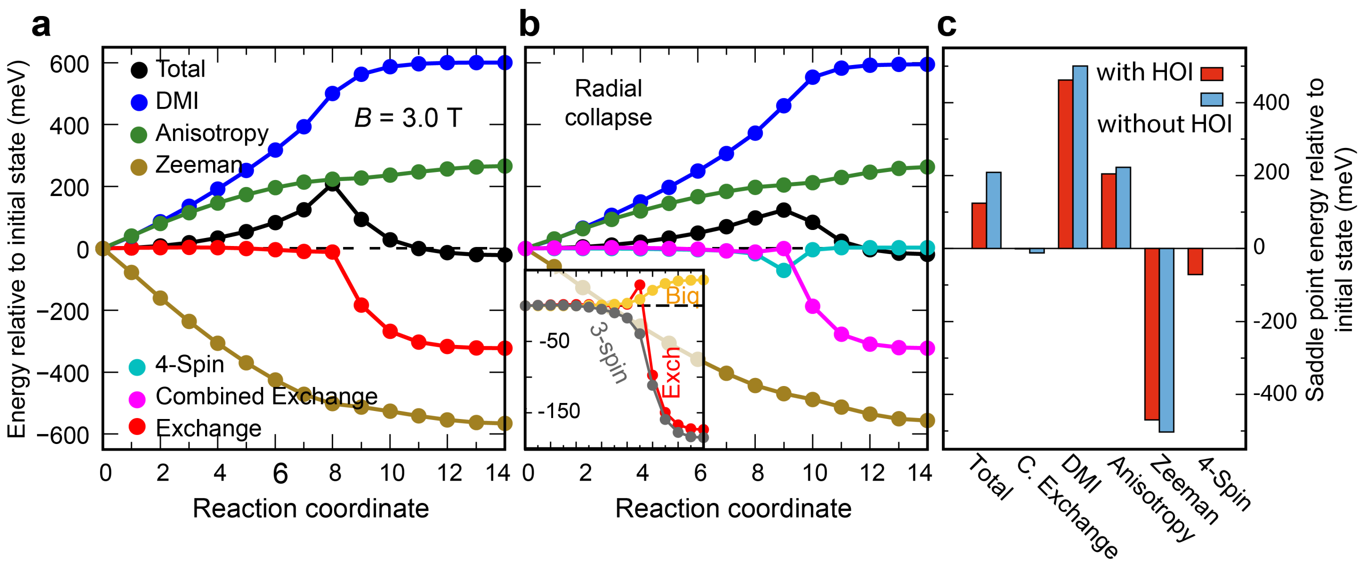

Supplementary Figure 4 | Minimum energy path of skyrmion collapse in Fe/Rh/Re(0001). Total and individual energy contributions without **a** and with **b** the HOI along minimum energy path. Both paths correspond to the radial collapse of isolated skyrmions. **c** Total and individual energy decomposition at the saddle point with respect to the initial state (skyrmion) without and with higher-order contributions. For brevity, the two-site, the three-site, and four-site four spin interactions are denoted as Biq, 3-spin and 4-spin, respectively.

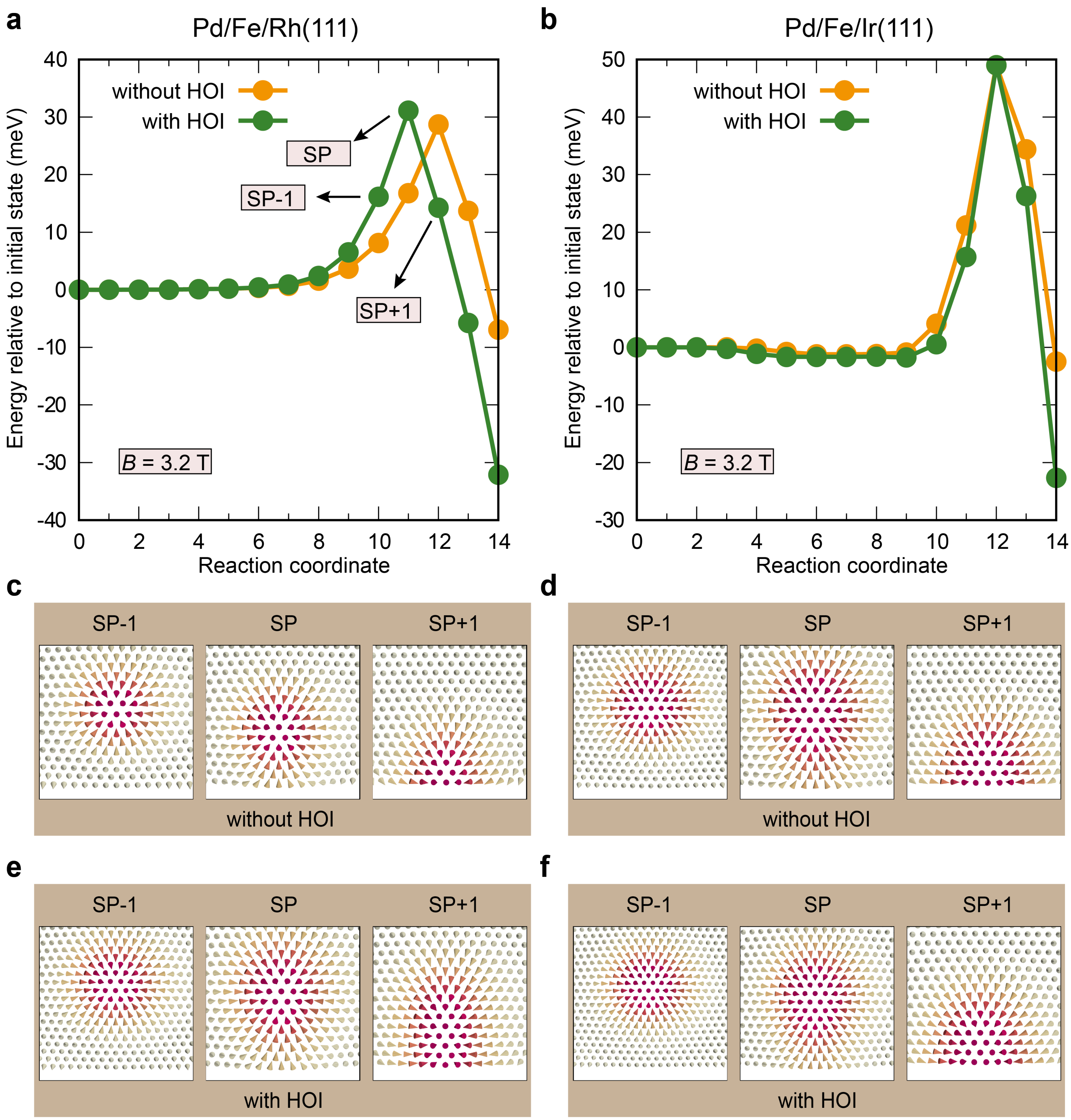

Supplementary Figure 5 | Energy barriers of skyrmions for escape through the boundary edges. a,b Energy barriers of isolated skyrmions with and without including HOI for escape through the boundary edges at $B = 3.2$ T for Pd/Fe/Rh(111) and Pd/Fe/Ir(111), respectively. **c,d** Spin structures before (SP-1), after (SP+1) and at the saddle point (SP) for escape through boundary without including HOI for Pd/Fe/Rh(111) and Pd/Fe/Ir(111), respectively. **e,f** Spin structures before (SP-1), after (SP+1) and at the saddle point (SP) for escape through boundary with including HOI for Pd/Fe/Rh(111) and Pd/Fe/Ir(111), respectively.

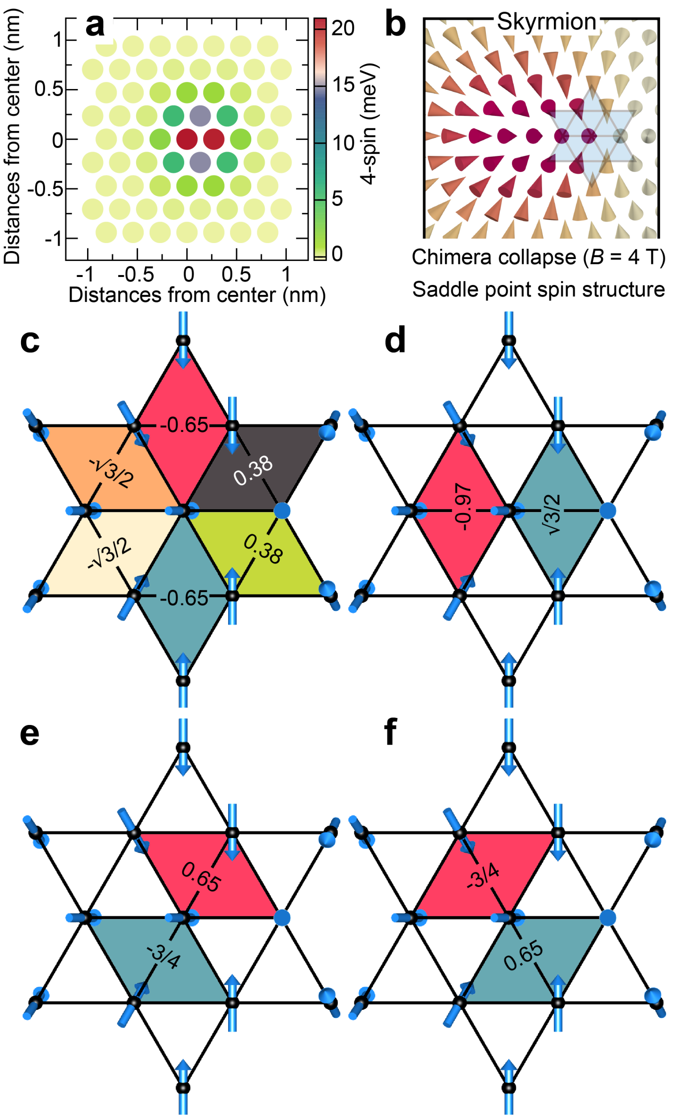

Supplementary Figure 6 | Four-site four spin energy at the chimera saddle for Pd/Fe/Ir(111). a Atomic-site resolved energy contribution of the four-site four spin interaction at the saddle points of the minimum energy paths of Supplementary Figure 3**b**, i.e., for the chimera skyrmion collapse. Energies are given with respect to the initial state. **b** Saddle point spin structure of Supplementary Figure 3**b**. **c-f** Spin structure around the core spin at the origin, i.e., at $(0, 0)$, of the saddle point is highlighted in **b** by the gray shaded star. The sign and the value of the four-site four spin term is shown for all twelve contributing diamonds. The total four-site four spin energy contribution amounts to $-2.58K_1$. For brevity, the four-site four spin interaction is denoted as 4-spin in **b**.

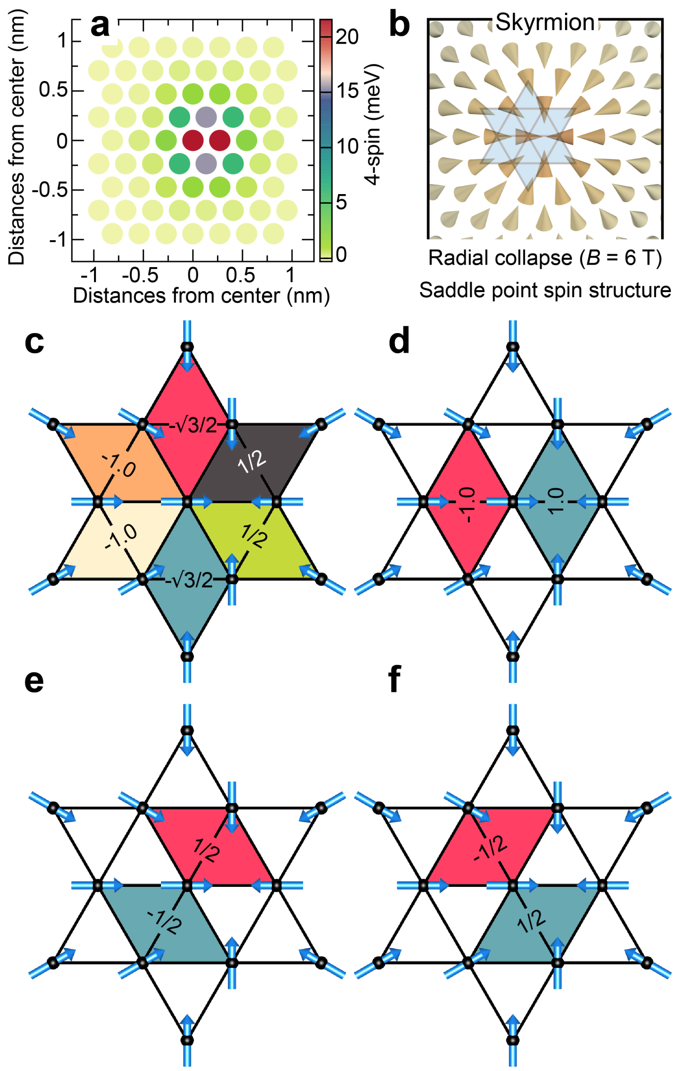

Supplementary Figure 7 | Four-site four spin energy at the saddle point of the radial skyrmion collapse for Pd/Fe/Ir(111). **a** Atomic-site resolved energy contribution of the four-site four spin interaction at the saddle points of the minimum energy paths of Supplementary Figure 3**e**, i.e. for the radial skyrmion collapse. Energies are given with respect to the initial state. **b** Saddle point spin structure of Supplementary Figure 3**e**. **c-f** Spin structure around the core spin at the origin, i.e., at $(0, 0)$, of the saddle point is highlighted in **b** by the gray shaded star. The sign and the value of the four-site four spin term is shown for all twelve contributing diamonds. The total four-site four spin energy contribution amounts to $-2.73K_1$. For brevity, the four-site four spin interaction is denoted as 4-spin in **b**.

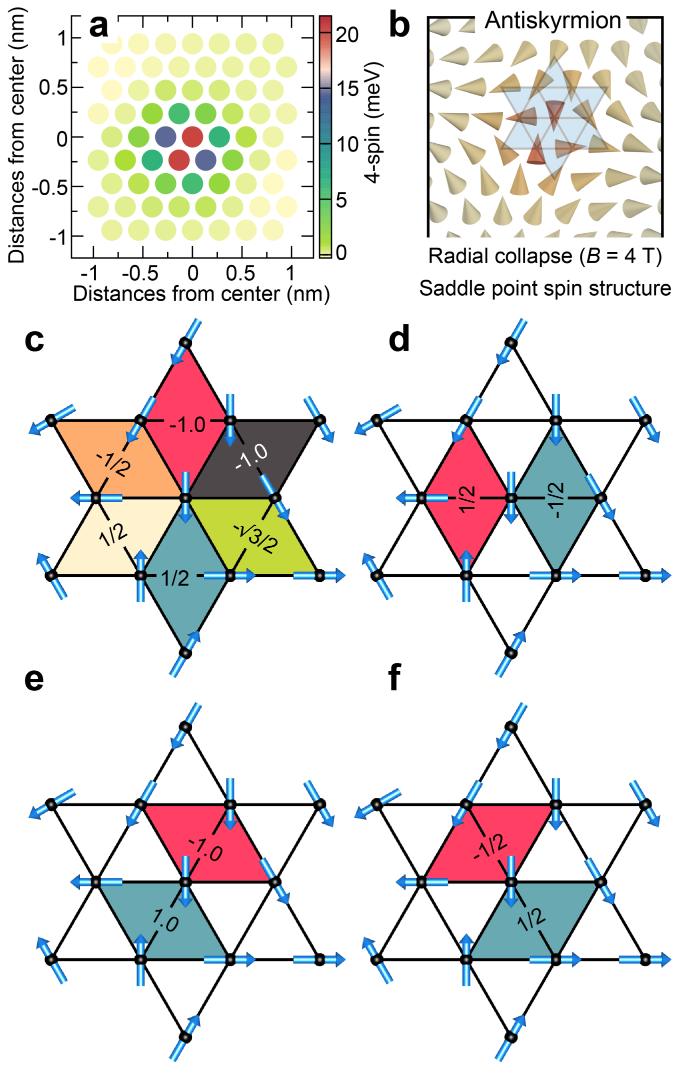

Supplementary Figure 8 | Four-site four spin energy at the saddle point of the antiskyrmion collapse for Pd/Fe/Ir(111). a Atomic-site resolved energy contribution of the four-site four spin interaction at the saddle points of the minimum energy paths of Supplementary Figure 3**h**, i.e., for the antiskyrmion collapse. Energies are given with respect to the initial state. **b** Saddle point spin structure of Supplementary Figure 3**h**. **c-f** Spin structure around the core spin at the origin, i.e., at $(0, 0)$, of the saddle point is highlighted in **b** by the gray shaded star. The sign and the value of the four-site four spin term is shown for all twelve contributing diamonds. The total four-site four spin energy contribution amounts to $-2.37K_1$. For brevity, the four-site four spin interaction is denoted as 4-spin in **b**.

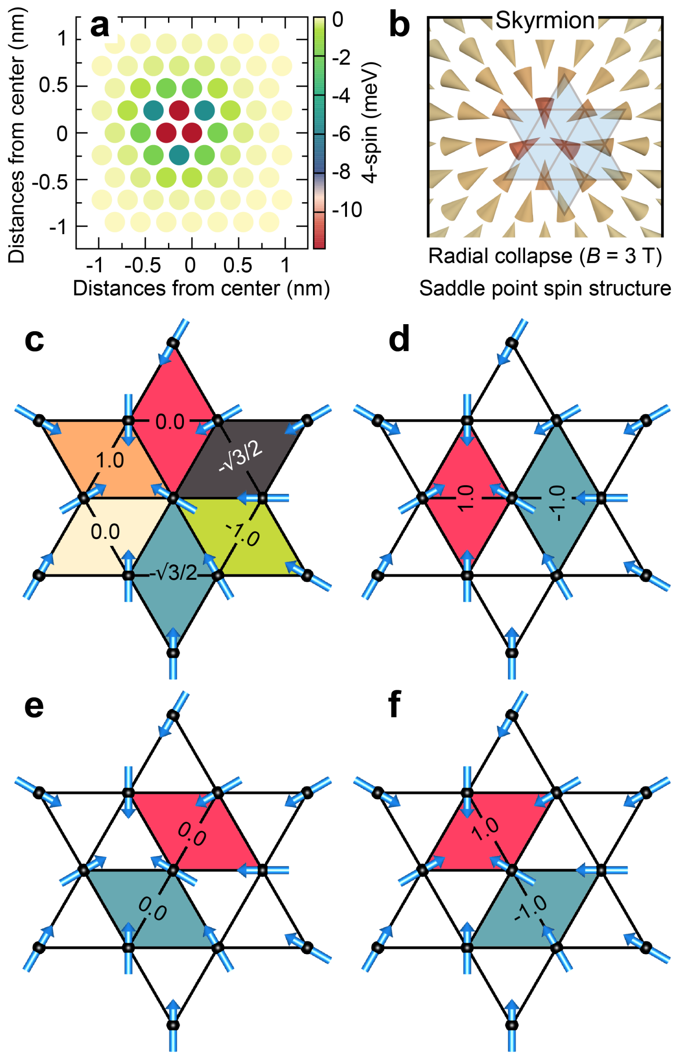

Supplementary Figure 9 | Four-site four spin energy at the saddle point of the skyrmion collapse for Fe/Rh/Re(0001). **a** Atomic-site resolved energy contribution of the four-site four spin interaction at the saddle points of the minimum energy paths of Supplementary Figure 4**b**, i.e., for the radial skyrmion collapse. Energies are given with respect to the initial state. **b** Saddle point spin structure of Supplementary Figure 4**b**. **c-f** Spin structure around the core spin at the origin, i.e., at $(0, 0)$, of the saddle point. The sign and the value of the four-site four spin term is shown for all twelve contributing diamonds. The total four-site four spin energy contribution amounts to $-1.73K_1$. For brevity, the four-site four spin interaction is denoted as 4-spin in **b**.

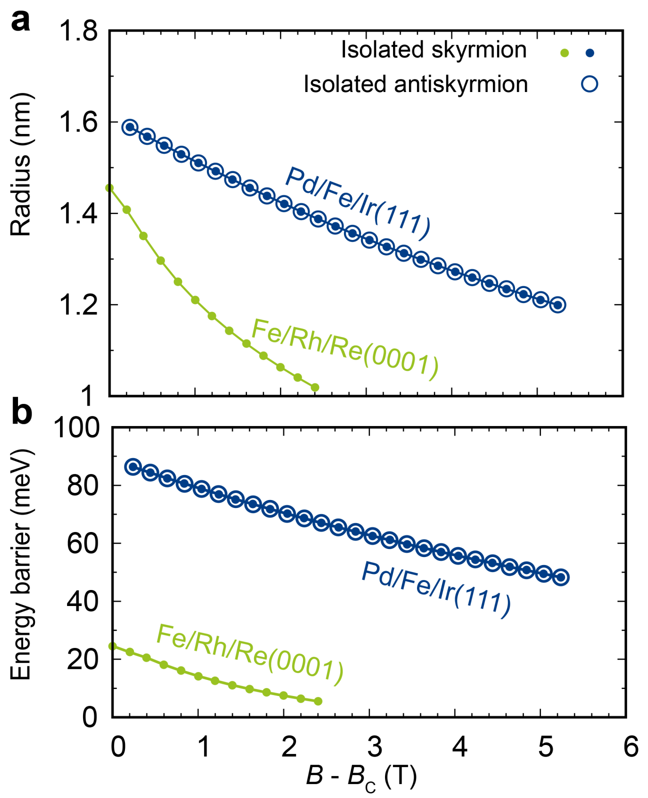

Supplementary Figure 10 | Radius and energy barriers of skyrmions or antiskyrmions including HOI at vanishing DMI. a,b Radius and energy barriers of isolated skyrmions and antiskyrmions including HOI but setting DMI to zero, respectively. Higher-order exchange constants obtained from DFT are used for Pd/Fe/Ir(111) (cf. Table **2**). For Fe/Rh/Re(0001), the biquadratic (B_1) and three-site four spin interaction (Y_1) constants from DFT were used. A four-site four spin interaction constant (K_1) with value of $K_1 = +1.36$ meV has been used, i.e., the sign has been inverted with respect to the DFT value in order to stabilize skyrmions and antiskyrmions.

Supplementary References

[1] Haldar, S., von Malottki, S., Meyer, S., Bessarab, P. F. & Heinze, S. First-principles prediction of sub-10-nm skyrmions in Pd/Fe bilayers on Rh(111). *Phys. Rev. B* **98**, 060413 (R) (2018).

[2] von Malottki, S., Dupé, B., F. Bessarab, P., Delin, A. & Heinze, S. Enhanced skyrmion stability due to exchange frustration. *Sci. Rep.* **7**, 12299 (2017).

[3] Paul, S. & Heinze, S. Tailoring magnetic interactions in atomic bilayers of Rh and Fe on Re(0001). *Phys. Rev. B* **101**, 104408 (2020).

[4] Huang, S. X. & Chien, C. L. Extended skyrmion phase in epitaxial FeGe(111) thin films. *Phys. Rev. Lett.* **108**, 267201 (2012).

[5] Banerjee, S., Rowland, J., Erten, O. & Randeria, M. Enhanced stability of skyrmions in two-dimensional chiral magnets with Rashba spin-orbit coupling. *Phys. Rev. X* **4**, 031045 (2014).

[6] Lin, S.-Z., Saxena, A. & Batista, C. D. Skyrmion fractionalization and merons in chiral magnets with easy-plane anisotropy. *Phys. Rev. B* **91**, 224407 (2015).

[7] Vousden, M. *et al.* Skyrmions in thin films with easy-plane magnetocrystalline anisotropy. *Appl. Phys. Lett.* **108**, 132406 (2016).

[8] Leonov, A. O. & Kézsmárki, I. Skyrmion robustness in noncentrosymmetric magnets with axial symmetry: The role of anisotropy and tilted magnetic fields. *Phys. Rev. B* **96**, 214413 (2017).

[9] Hayami, S. & Motome, Y. Effect of magnetic anisotropy on skyrmions with a high topological number in itinerant magnets. *Phys. Rev. B* **99**, 094420 (2019).
