# Supplementary material for: Role of higher-order exchange interactions for skyrmion stability
Source: Nat Commun. 2020 Sep 21;11:4756. doi: 10.1038/s41467-020-18473-x (PMC7506016; doi:10.1038/s41467-020-18473-x)
Supplement: Supplementary file 2 — Description of Additional Supplementary Files [file 41467_2020_18473_MOESM2_ESM.pdf]

Description of Additional Supplementary Files

Title: Supplementary Data 1

Description: The exchange and HOI parameters from DFT of Supplementary Table 1 with the full number of digits used in the atomistic spin dynamics simulations.

Title: Supplementary Data 2

Description: The Dzyaloshinskii-Moriya interactions parameters, the magnetocrystalline anisotropy parameter as well as the magnetic moments from DFT of Supplementary Table 3 with the full number of digits used in the atomistic spin dynamics simulations
